# Impact of Cetyl-Containing Ionic Liquids on Metal Halide Perovskite Structure and Photoluminescence

**DOI:** 10.3390/nano15130964

**Published:** 2025-06-21

**Authors:** Maegyn A. Grubbs, Roberto Gonzalez-Rodriguez, Sergei V. Dzyuba, Benjamin G. Janesko, Jeffery L. Coffer

**Affiliations:** 1 Department of Chemistry and Biochemistry, Texas Christian University, TCU Box 298860, Fort Worth, TX 76129, USA; m.grubbs@tcu.edu (M.A.G.); s.dzyuba@tcu.edu (S.V.D.); b.janesko@tcu.edu (B.G.J.); 2 Department of Physics, University of North Texas, Denton, TX 76203, USA; roberto.gonzalezrodriguez@unt.edu

**Keywords:** perovskites, ionic liquids, defect passivation, light emitting diode

## Abstract

Ionic liquids (ILs) can ideally reduce defects and improve the film stability of emissive metal halide perovskite films. In this work, we measure how the structure and emission of methylammonium lead tribromide (MAPbBr_3_) perovskite films is modulated by long alkyl chain-containing pyridinium, imidazolium, or pyrrolidinium ILs. Two different film deposition methods are compared, with the resultant films characterized by X-ray diffraction (XRD), scanning electron microscopy (SEM), and photoluminescence (PL) spectroscopy. For the latter, the differences in PL intensity of the perovskite are quantified using photoluminescence quantum efficiency (PLQE) measurements. It is found that a spin coating method in conjunction with the use of an imidazolium-containing IL (for a given precursor concentration) produces the strongest emissive perovskite. This optimal enhancement is attributed to a function of accessible surface charges associated with the heterocyclic cation of a given IL and perovskite defect passivation by bromide, the latter elucidated with the help of density functional theory. Proof-of-concept device fabrication is demonstrated for the case of a light emitting diode (LED) with the IL present in the emissive perovskite layer.

## 1. Introduction

Extensive studies of metal halide perovskites (MHPs) in photovoltaics and light emitting diodes (LEDs) come in part from their fundamental electronic properties such as low exciton binding energies/high charge carrier mobility resulting in diffusion across long distances in a crystal [1,2,3]. In evaluating the relative quality of these perovskites, one fundamental property of significance is photoluminescence quantum efficiency (PLQE) [4,5,6,7,8]. In spite of defect tolerance, in order to produce efficient films (ideally near 100% PLQE), the maximization of radiative recombination and the minimization of nonradiative recombination are required by controlling defects in the crystalline structure and film interface.

While perovskites are easily fabricated, their crystal size and number of defects present are challenging to control. Vacancies, site substitutions, and halide segregation can lead to surface, intercrystalline, and intracrystalline defects which can result in nonradiative recombination or trap states [9]. One approach to reduce the number of defects and improve film stability is to use ionic liquids (ILs) during perovskite formation. ILs are organic salts made typically of organic cations and inorganic (or organic) anions and possess melting points below 100 °C [10,11]. Ionic liquids have been extensively used in earlier studies to direct and influence the properties of selected nanostructures [12]. For the particular case of perovskites, they can be added to the perovskite precursor solution to slow down the crystallization process so that ideally fewer defects and vacancies are created [13]. ILs can also be applied to the antisolvent layer (ASs), solvents that do not dissolve perovskites or their precursors, to reduce defects in film morphology [14,15]. Due to their non-volatile nature, the ILs will be present in films after crystallization. Thus, ILs, used as additives, depending on cation/anion identity, can facilitate perovskite formation [10,16].

**Figure 1:**
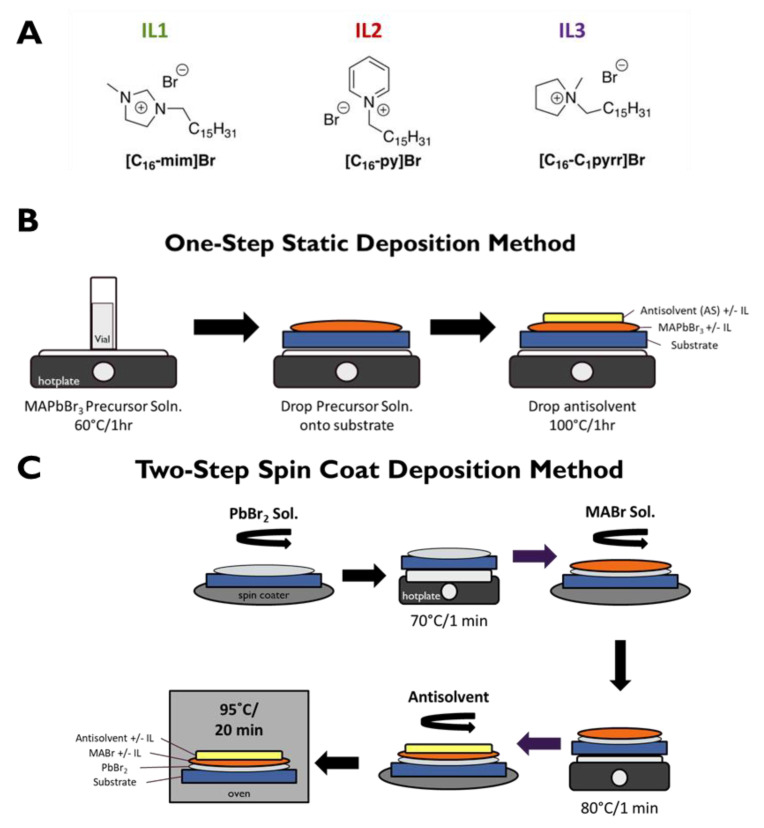
(**A**) Structures of ILs used in these experiments. (**B**) One-step static deposition method. (**C**) Two-step spin coat deposition method. The substrate is typically either FTO or Si.

A number of ILs have been used as additive/components of perovskite-based materials and devices [17,18,19], yet the application of designer solvent capabilities of ILs in such devices remains largely underexplored, even though it holds great potential for tuning and enhancing device performance [10,20,21]. Therefore, as an initial entry of exploring how the structure of ILs could impact the structure of perovskite-based materials, here we investigate selected long alkyl chain-containing ILs based on several heterocyclic cations. Specifically, cetyl (n-hexadecyl alkyl chain) ILs are investigated for their effects on methylammonium lead tribromide (MAPbBr_3_) perovskite morphology and photoluminescence (PL). The three ILs studied here are [C_16_-mim]Br (an imidazolium IL, referred to as “I**L1**”), [C_16_-py]Br (a pyridinium IL, referred to as “**IL2**”), and [C_16_-C_1_pyrr]Br/, (pyrrolidinium IL, referred to as “**IL3**”) (Figure 1A). The choice of these ILs has been driven by the ability of such ILs to control structural features of various nanomaterials [22,23,24,25], including those that feature perovskites [26,27]. It should also be noted that **IL1–IL3** have been used as surfactants [28,29,30], bioactive agents [31,32,33,34], and gelators [35] of various types of fluids.

Previous studies have tested the effects of alkyl-group-containing (albeit with nine or fewer carbons) imidazolium and pyrrolidinium ILs with different anions on the formation of perovskite films [10,16]. It is hypothesized that the inclusion of a longer (such as cetyl) alkyl chain-containing ILs will protect the perovskite films from the environment by increasing hydrophobicity [36,37] and constraining crystal growth/increasing crystal nucleation, leading to smaller grain sizes and ideally fewer defects in the grain boundaries. Additionally, ILs can help improve film morphology, specifically pinholes. Defect passivation can occur via the Br^–^ of the IL interaction with Br and Pb vacancies or migration throughout the perovskite film. This combination of effects will ideally minimize nonradiative recombination.

Multiple methods for metal halide perovskite film fabrication exist [38,39]; this project specifically compares two deposition methods, a one-step static method and a two-step spin coat route [39]. In addition to evaluating the effects of these three cetyl-containing ILs on the structure and properties of MAPbBr_3_ perovskites, the location of IL addition during perovskite film fabrication and the concentration of the ILs were also tested. Antisolvent (AS) was also used to improve crystal surface morphology by helping eliminate macroscopic pinhole defects [15]. Limiting the trapping of charge carriers and reducing the self-absorption of photons were achieved by decreasing the grain sizes and film thickness [9]. Computation using density functional theory (DFT) was used to assist in our understanding of the role of the cation of the IL on perovskite PL efficiency. Finally, the feasibility of using these specific ILs in practical device platforms is also demonstrated for the case of a simple LED configuration.

## 2. Materials and Methods

Materials. Materials used include lead (II) bromide (>98.0%, TCI, Tokyo, Japan), methylammonium bromide (>98.0%, TCI), dimethylformamide (99.8%, Alfa Aesar, Ward Hill, MA, USA), dimethyl sulfoxide (>99.0%, TCI, Tokyo, Japan), toluene (ACS grade, Pharmco, Shelbyville, KY, USA), and ethanol (200 proof, Pharmco, Shelbyville, KY, USA). The three cetyl-containing ILs were synthesized according to procedures outlined in the literature [35].

Instrumentation. Photoluminescence measurements were performed using an Ocean Optics S-2000 spectrometer (Largo, FL, USA) interfaced with a Nikon Optiphot fluorescent microscope (Tokyo, Japan) containing a mercury lamp and an excitation filter centered at 370 nm. Scanning electron microscopy (SEM) images were obtained using a JEOL JSM-7100F with an energy dispersive X-ray analyzer (EX-230) (Tokyo, Japan). X-ray diffraction was performed on a Rigaku Smartlab SE (Woodlands, Texas, USA) with a Cu X-ray source. Photoluminescence quantum efficiency (PLQE) measurements were performed with a 405 nm laser diode as exciting light, a neutral density filter, and an integrated sphere setup according to Leyre et al. [40]; the setup was verified using barium magnesium aluminate doped with europium (BAM) as a standard (96 ± 2%) [40,41].

Computation. The computed electrostatic potential (ESP) of IL1–IL3 head groups used calculations that treat the isolated IL cation and replace the cetyl chain with ethyl. Calculations use density functional theory (DFT) with the B3LYP exchange-correlation functional [42,43,44,45], the def2TZVP basis set [46], and the SMD continuum model for acetonitrile solvent [47]. Molecular geometries are optimized at this level of theory. ESP are reported on the 0.001 electrons/bohr^3^ density iso-surfaces [48]. Calculations are performed in the Gaussian 16 package [48].

Film Preparation—One-Step Static Method (Figure 1B*)*. In total, 0.8 M MAPbBr_3_ precursor solution was made by dissolving lead bromide and methylammonium bromide at equimolar concentrations in DMF. For MAPbBr_3_ + IL (in MABr and MABr/AS) films, 300 µL of the precursor solution was put into an empty vial and 30 mol% of IL was added. IL was also added to the AS for the MAPbBr_3_ + IL (in AS and MABr/AS films). The solutions were then heated at 60 °C on a hotplate for 1 h. Lastly, the precursor solutions were added dropwise (total of ~50 µL) onto a given substrate, toluene antisolvent was added dropwise on top (total of ~50 µL), and the samples were heated on a hotplate at 100 °C for 1 h. The evaluation of perovskite PL intensity as a function of IL concentration in the 0–30 mol% range determined that for a given IL, optimal PL intensity was produced with the highest IL concentration (Figure S1). Note that we did not attempt to employ IL concentrations above 30 mol% so as to avoid IL solubility limitations as well as the possible formation of two-dimensional (2D) perovskite phases.

Film Fabrication—Two-Step Spin Coat Method (Figure 1C). A 1.5 M PbBr_2_ solution was made with PbBr_2_ and KBr in DMF: DMSO (9:1 *v*/*v*). A 0.5 M MABr solution was made with MABr in absolute ethanol. The above PbBr_2_ solution (100 µL) was added dropwise onto the substrate, spun at 3000 rpm for 30 s, then heated at 70 °C for 1 min. Next, the MABr solution (100 µL) was added dropwise on top of the PbBr_2_ layer, spun at 3000 rpm for 30 s, then heated at 80 °C for 1 min. The films were washed with 25 µL of absolute ethanol and spin-coated, 25 µL of antisolvent was added to the top layer, and the solution was again spun at 3000 rpm for 30 s and lastly heated at 95 °C for 20 min. For the MAPbBr_3_ + IL films, IL was added in different permutations: (1) to the MABr solution alone, (2) to the antisolvent alone, or (3) to both. It is found that the precise location of the IL is not a significant factor in optimizing PL intensity, as long as it is present during film formation (due presumably to IL diffusion in the film) (Figure S2).

## 3. Results and Discussion

**Figure 2:**
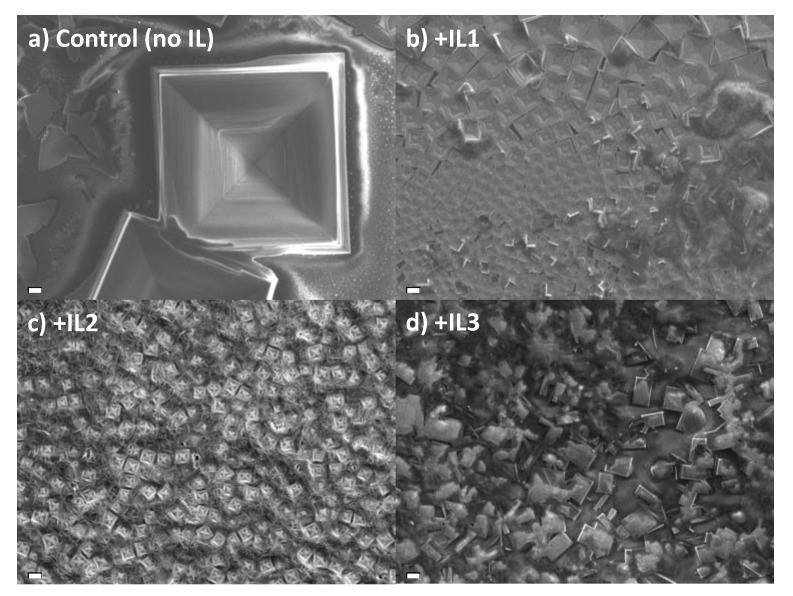
SEM images of MAPbBr_3_ thin films prepared by a one-step static method with (**a**) no IL added, (**b**) **IL1**, (**c**) **IL2**, and (**d**) **IL3**. Scale bar—10 µm.

Static One-step Method. We begin with an examination of the influence of ILs on film morphology under static conditions for the purpose of allowing the perovskite to crystallize under conditions whereby the IL is presumably allowed to retain its equilibrium geometry in film formation. The SEM images (Figure 2) show morphological differences between the perovskite films made with **IL1**, **IL2**, and **IL3** using this static method, along with the MAPbBr_3_ prepared in the absence of IL. All four films show the presence of a macroscopic cube shape characteristic of cubic MAPbBr_3_, but the presence of IL has a significant impact on cube size. When compared to the control film (no IL), the cube size decreases from the IL-absent control of ~180 μm to ~11–15 μm when IL is present. The cubes of the control, +**IL1**, and +**IL2** films show distinct faceting on their surfaces while the film with **IL3** has smooth surfaces.

The X-ray diffraction (XRD) patterns of each sample possess the characteristic <100> and <200> peaks consistent with the cubic phase of MAPbBr_3_ (Figure S3) [49,50]. The quantitative analysis of perovskite crystallite size using a Halder–Wagner-type method reveals relatively larger crystallites for MAPbBr_3_ prepared with **IL1** but significantly smaller grains using **IL2** or **3** (Table 1). In any event, the crystallite domain size assessed by XRD is far smaller (70–100 nm) than the feature size determined by direct SEM imaging, as noted above.

**Figure 3:**
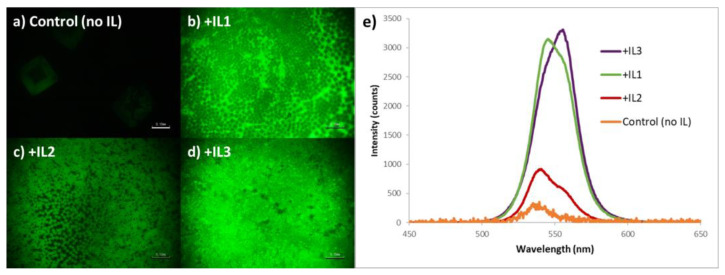
Photoluminescent images of MAPbBr_3_ thin films prepared via a one-step static method with (**a**) no IL added, (**b**) + **IL1**, (**c**) + **IL2**, and (**d**) + **IL3**, each at 30 mol% (*w*/*w*) (scale bar = 100 mm); (**e**) corresponding PL spectra of the above films (note that the intensity of the non IL-containing sample, i.e., control, is ×10).

While sharing a common morphology, there are distinct differences in emission intensity of the MAPbBr_3_ as a function of IL identity. The relatively large-area PL imaging of these samples reveals that for the static one-step deposition method, the control film (no IL) was effectively non-emissive under the conditions utilized, and it is also clear that the perovskite film containing MAPbBr_3_ + **IL2** has the weakest emission intensity of the three ionic liquid-containing films (Figure 3a–d). This observation correlates with the measured PL spectra. As seen in Figure 3e, MAPbBr_3_ + **IL3** perovskite films consistently demonstrated the highest emission intensity, MAPbBr_3_ + **IL2** perovskite films the lowest intensity, with values for MAPbBr_3_ + **IL1** lying close to that of the **IL3**-containing films.

PLQE measurements of the MAPbBr_3_ +IL films utilizing an integrated sphere found that **IL3**-containing films have a PLQE value of 9.6%, **IL1**-containing films 3.7%, and both **IL2** and control films are beyond the lower limit of detection with our system.

The nature of the perovskite crystallization process associated with this method infers a preference of the five-membered ring heterocycles (imidazolium and pyrrolidinium) over the six-membered (pyridinium) moiety. MAPbBr_3_ sampled prepared in the presence of **IL1** and **IL3** are both relatively brighter than the others, with **IL3** being slightly higher with regard to PLQE; yet for each, the PL is relatively broad and shows the clear spectroscopic signature of Br vacancies (560 nm shoulder) [51].

Two-Step Spin Coat Method. The likely self-absorption/photon recycling events and associated low PLQE values of the relatively thick perovskite films formed in the presence of these cetyl-containing ILs under static conditions led us to investigate two-step spin coating deposition methods for the purpose of producing thinner films and ideally decreasing the amount of bromine vacancies.

**Figure 4:**
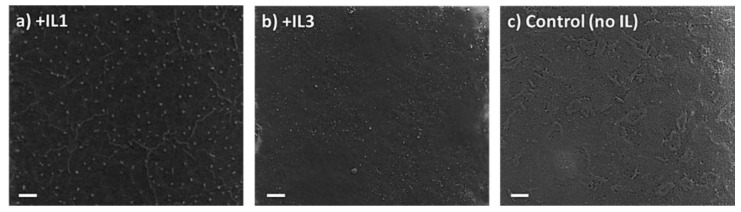
SEM images of MAPbBr_3_ thin films prepared via a two-step spin coat method with (**a**) +**IL1** in MABr and AS layers; (**b**) +**IL3** in MABr and AS layers; (**c**) no IL added. Scale bar—10 µm.

The XRD patterns of the perovskite films +/− IL deposited through the two-step spin coat method show the same <100> and <200> peaks seen in the static one-step method, again consistent with a cubic crystal structure (Figure S4). Morphologically, however, in contrast to the one-step static method, no macroscopic cubes are seen in the SEM images of the two-step spin coat films, but rather continuous films are observed which have an absence of distinct crystal domains and boundaries for both the MAPbBr_3_ film with and without the addition of IL (Figure 4). The quantitative analysis of the X-ray diffraction linewidths indicates comparable crystallite grain sizes for all perovskites formed by this method (Table 2). It should be noted that the morphology of the control (i.e., no IL) films produced by the two-step spin coat deposition method is pitted while the films with **IL1** or **IL3** are not. The thickness of the MAPbBr_3_ + **IL1** films was measured using a SEM cross-sectional image shown in Figure S5 with an average thickness of 270 nm. Energy-dispersive X-ray spectroscopy (EDX) was performed on the perovskite films with and without IL (Figure S6). As anticipated, the perovskite films with **IL1** or **IL3** show an increase in mass percentage of carbon, nitrogen, and bromine compared to the control perovskite film (no IL). It should also be noted that the EDX spectra of the speck-like regions of the film possess similar values to the perovskite + **IL1** films where the film is continuous (no specks), i.e., confirming that these specks were not perovskite (Figure S6b).

**Figure 5:**
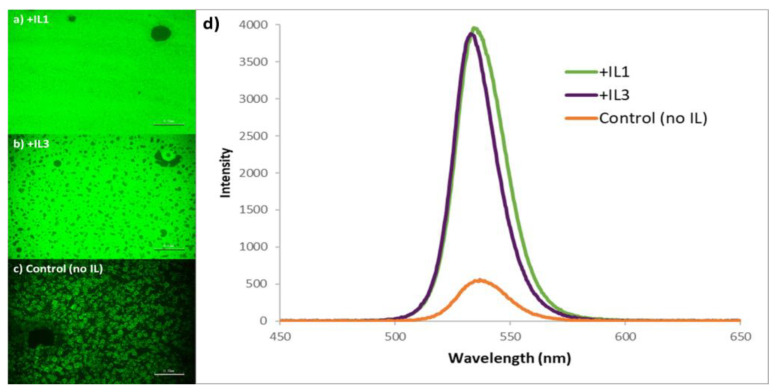
Photoluminescent images of MAPbBr_3_ thin films prepared using the two-step spin coat method with (**a**) **+IL1** in MABr and AS layers, (**b**) **+IL3** in MABr and AS layers, and (**c**) no IL added (scale bar: 0.10 mm). (**d**) Photoluminescence spectra of MAPbBr_3_ thin films +/− IL prepared using the two-step spin coat method. Spectra for MAPbBr_3_ stabilized by **IL1** and **IL3** are normalized to highlight differences with the non-IL-containing control.

Spectroscopically, there is a marked improvement in both PL intensity and line shape using this two-step method. In addition to the large-area PL images demonstrating relatively brighter and more uniform emission in the case of MAPbBr_3_ + **IL3**, the emission maxima are now at 535 nm, with a relatively narrower full width at half maximum (FWHM); the significant reduction in emission intensity of the 560 nm shoulder is consistent with the observation that bromine vacancies were reduced in the films produced using this method (Figure 5d) [51]. The two-step spin coat deposition method also resulted in films having much higher PLQE values than the one-step static method; films with **IL3** consistently measured ~30% in contrast to the control films (without IL) that reached ~5%.

The PLQE of the samples containing **IL1** prepared via the two-step spin coat deposition method was evaluated next, again with relatively brighter emission and the PLQE of the perovskite improving to 50–70% (significantly higher than the 4% value associated with the perovskite prepared using **IL1** via the one-step method).

**Figure 6:**
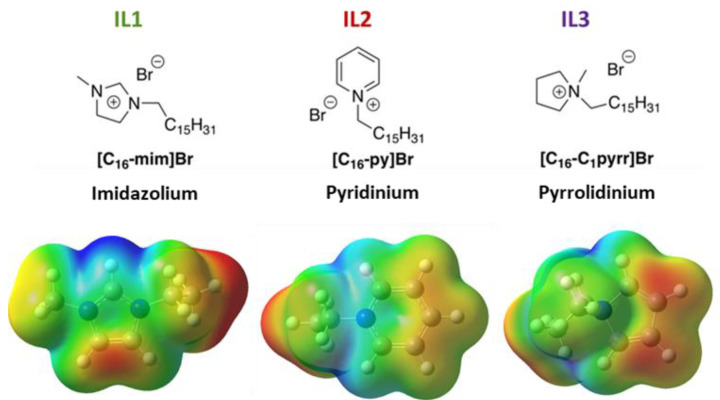
Structures of three ILs used in this study, along with computed electrostatic potential maps. Red denotes least positive surface area (+0.13 au) and blue denotes most positive (+0.2 au). In these calculations, cetyl chains are replaced with ethyl groups for computational efficiency.

To provide better insight into the greater efficiency of perovskite films containing **IL1** than those made with **IL3**, we turned to DFT calculations of the three respective ILs (Figure 6). This figure shows the computed 0.001 electrons bohr^−3^ density iso-surface colored by computed electrostatic potential: red is least positive (+0.13 au) and blue is most positive (+0.2 au). To accelerate the calculations, the cetyl group was replaced with the ethyl group. The most positive region of all three ILs is the imidazolium H on carbon 2 of **IL1**. We hypothesize that the electrostatics of the bromide associated with a given ionic liquid drive its passivation ability. If more optimal packing of bromide at the perovskite surface is present for the imidazolium-containing IL, and the larger pyridinium moiety of IL2 is the worst, then this effect could explain the relative order of PLQE values for these IL-containing perovskite films.

How is this the case? In long-chain ionic liquids, it is well known that electrostatics (that is, the coulombic force associated with the charged ion pair) is the driving force behind the diffusion of the IL in solution [52]. If the passivation of bromide vacancies drives enhanced PL in general, then the passivation by IL should be driven by the ability of the IL to transfer bromide ions to the growing perovskite film. The strong ion pairing between bromide and the highly charged imidazolium would explain the possible behavior of **IL1** given its computed greater positive charge. The spin coating of the ionic liquid during perovskite formation also serves to further accelerate the process of defect passivation and PL enhancement in a dynamic fashion, as noted by the outcomes in a comparison of Methods 1 and 2. This effect does not explain differences between **IL2-** and **IL3**-induced behavior, however, suggesting that the cetyl chain plays more of a role in the case of perovskite formed in the presence of these two ILs.

Light-Emitting Diode (LED) Fabrication. To assess the feasibility of cetyl-containing ILs embedded in perovskite device configurations, the optimized MAPbBr_3_ + **IL1** film prepared via a two-step spin coat method was used as the emissive layer in a simple light emitting diode (LED).

**Figure 7:**
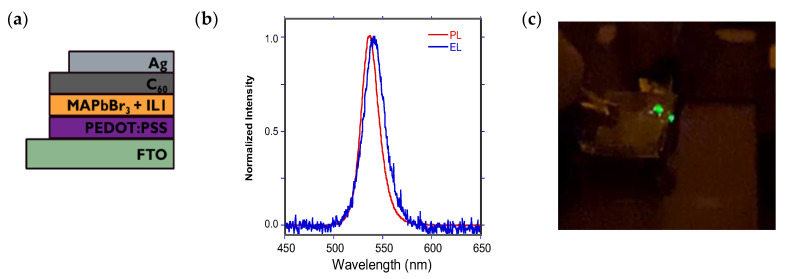
(**a**) Device stack diagram for a MAPbBr_3_ + **IL1** configuration; (**b**) normalized EL and PL spectra for this LED; (**c**) green emission emanating from this device at 6 V bias.

A typical design is illustrated in Figure 7a. In addition to the MAPbBr_3_ + **IL1** emissive layer, PEDOT:PSS is employed as a hole transport layer, C_60_ as an electron transport layer, and Ag/FTO for electrical contact purposes [9,53]. While the current flow through the system is quite limited at 30 mol % IL (not surprisingly), the current–voltage curve does show proper rectification (Supporting Figure S7) with an onset voltage of 4 V, and green electroluminescence (EL) can be readily observed at 6 V with an emission maximum at 540 nm, red-shifted slightly from the PL max at 535 nm (Figure 7b); the EL is bright enough at this voltage to be visible with the unaided eye (Figure 7c). However, attempts to assess the external quantum efficiency (EQE) of such diodes using a calibrated photodiode resulted in values beyond the detection limit of our system (<0.02%).

## 4. Conclusions

This study examined the role of charged cationic head group identity in selected cetyl ionic liquids on the structure and PL of MAPbBr_3_ thin films. By examining the impact of both cation identity—pyridinium, imidazolium, or pyrrolidinium—and the film deposition route on perovskite structure and properties, we were able to optimize the PLQE of the MAPbBr_3_ formed in the presence of these ILs to values as high as 70%.

For reasons presumably related to electrostatics, imidazolium-containing ILs could be optimal additives for defect passivation and film structure. Further work is required to produce compositions and conditions necessary for useful device configurations. These and additional experiments are under consideration in our laboratories.

## Figures and Tables

**Table 1:** Crystallite size of MAPbBr_3_ samples prepared using Method 1 and analyzed using a Halder–Wagner-type method of powder XRD linewidths.

*Sample*	Crystallite Size (nm)
*One-Step Static Method*	
*Control (no IL)*	114.9 (4.1)
***+IL1***	124.5 (2.8)
***+IL2***	84.9 (1.4)
***+IL3***	77.0 (1.3)

**Table 2:** Crystallite size of MAPbBr_3_ samples prepared using Method 2 and analyzed according to a Halder–Wagner-type analysis of powder XRD linewidths. We focus here on perovskites formed with IL1 and IL3 due to their superior PL properties.

*Sample*	Crystallite Size (nm)
*Two-Step Spin Coat Method*	
*Control (no IL)*	85.5 (7.5)
***+IL1***	46.1 (7.6)
***+IL3***	52.3 (12.8)

## Data Availability

A copy of the original data is available from the authors upon request.

## Supplementary Materials

The following supporting information can be downloaded at: https://www.mdpi.com/article/10.3390/nano15130964/s1: Figure S1 (a) PL spectra of MAPbBr_3_ produced via one-step static method with varying mol% of **IL3**. SEM images of (b) MAPbBr_3_ + 30 mol% **IL3**; (c) + 20 mol% **IL3**; (d) no **IL3**; Figure S2: MAPbBr_3_ prepared via a one-step static deposition method with **IL3** added in varying locations: (a) PL spectra of all samples; PL imaging of perovskite with IL3 added to (b) precursor solution; (c) antisolvent; (d) non-IL control; (e–g) corresponding SEM images. (scale bar = 100 μm); Figure S3: X-ray diffraction patterns of MAPbBr_3_ perovskite films with the three cetyl ILs used in this study, prepared via a one-step static deposition method; Figure S4: X-ray diffraction patterns of MAPbBr_3_ perovskite films with the three cetyl ILs used in this study, prepared via a spin coating-based two-step deposition method; Figure S5: Cross-sectional SEM image of MAPbBr_3_ + IL1 produced via two-step spin coat method; Figure S6: Energy-dispersive X-ray (EDX) spectra of (a) MAPbBr_3_; (b) MAPbBr_3_ +**IL1**—specks region, (c) MAPbBr_3_ +**IL1**—no specks region; Figure S7: Current–voltage curve for a typical LED of composition Ag/C_60_/MAPbBr_3_ + **IL1**/PEDOT:PSS/FTO.
