# Supplementary material for: Impact of Cetyl-Containing Ionic Liquids on Metal Halide Perovskite Structure and Photoluminescence
Source: Nanomaterials (Basel). 2025 Jun 21;15(13):964. doi: 10.3390/nano15130964 (PMC12250683; doi:10.3390/nano15130964)
Supplement: Supplementary file 1 [file nanomaterials-15-00964-s001.zip › nanomaterials-3667851-supplementary.pdf]

Supporting Information

Impact of Selected Cetyl Ionic Liquids on Metal Halide Perovskite Structure and Photoluminescence

*Maegyn A. Grubbs,[1] Roberto Gonzalez-Rodriguez,[2] Sergei V. Dzyuba,[1] Benjamin G. Janesko[1] and Jeffery L. Coffer[1]**

[1] Department of Chemistry and Biochemistry, Texas Christian University, TCU Box 298860, Fort Worth, Texas 76129, USA

[2] Department of Physics, University of North Texas, Denton, Texas, 76201 USA

Figure S1: a) photoluminescence spectra of $MAPbBr_3$ produced via one step static method with varying mol% of IL3. SEM images of: b) $MAPbBr_3$ + 30 mol% IL3; c) + 20 mol% IL3; d) no IL3.

Figure S2: $MAPbBr_3$ prepared via a one-step static deposition method with IL3 added in varying locations: a) PL spectra of all samples; PL imaging of perovskite with IL3 added to (b) precursor solution; (c) antisolvent; (d) non-IL control; (e-g) corresponding SEM images. (scale bar = 100 mm)

Figure S3: X-ray diffraction patterns of $MAPbBr_3$ perovskite films with the 3 cetyl ILs used in this study, prepared via a one-step static deposition method.

Figure S4: X-ray diffraction patterns of $MAPbBr_3$ perovskite films with the 3 cetyl ILs used in this study, prepared via a spin coating-based two-step deposition method.

Figure S5: Cross sectional SEM image of $MAPbBr_3$ + IL1 two-step spin coat method.

Figure S6: Energy Dispersive X-ray (EDX) spectra of: a) $MAPbBr_3$; b) $MAPbBr_3$ +IL1 “specks” region, c) $MAPbBr_3$ +IL1 - no specks region.

Figure S7: Current-Voltage curve for a typical LED of composition Ag /C_{60} / $MAPbBr_3$ + **IL1** / PEDOT:PSS/FTO.

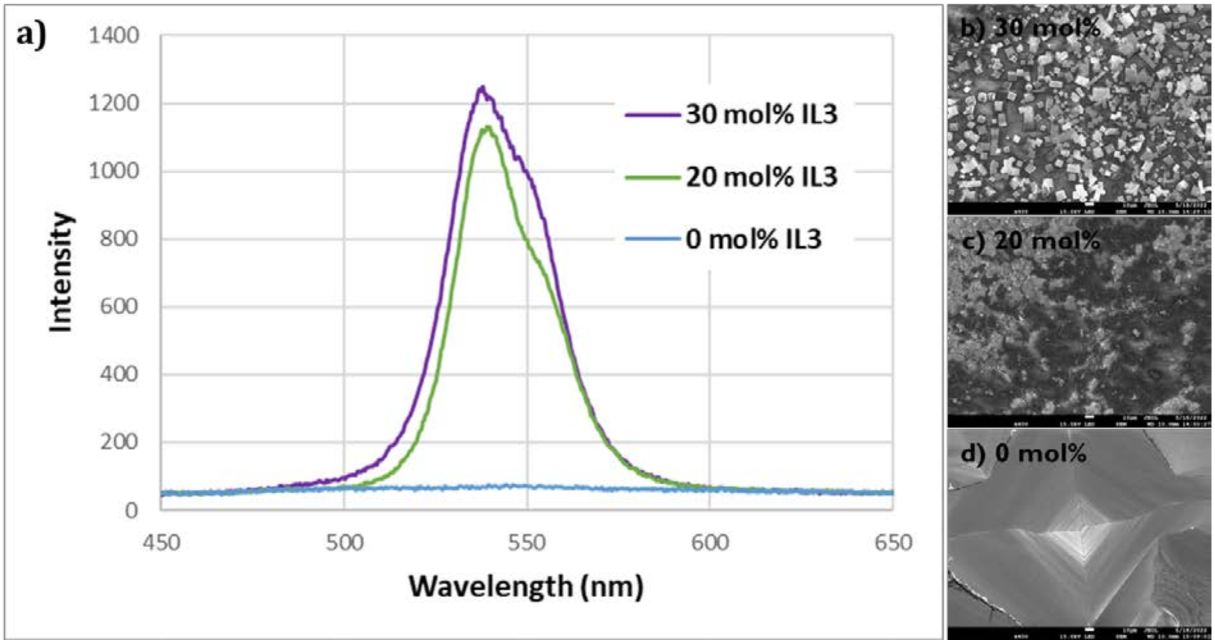

Figure S1. a) Photoluminescence spectra of $MAPbBr_3$ produced via the one step static method with varying mol% of IL3. SEM images of: b) $MAPbBr_3$ + 30 mol% IL3; c) + 20 mol% IL3; d) no IL3.

.

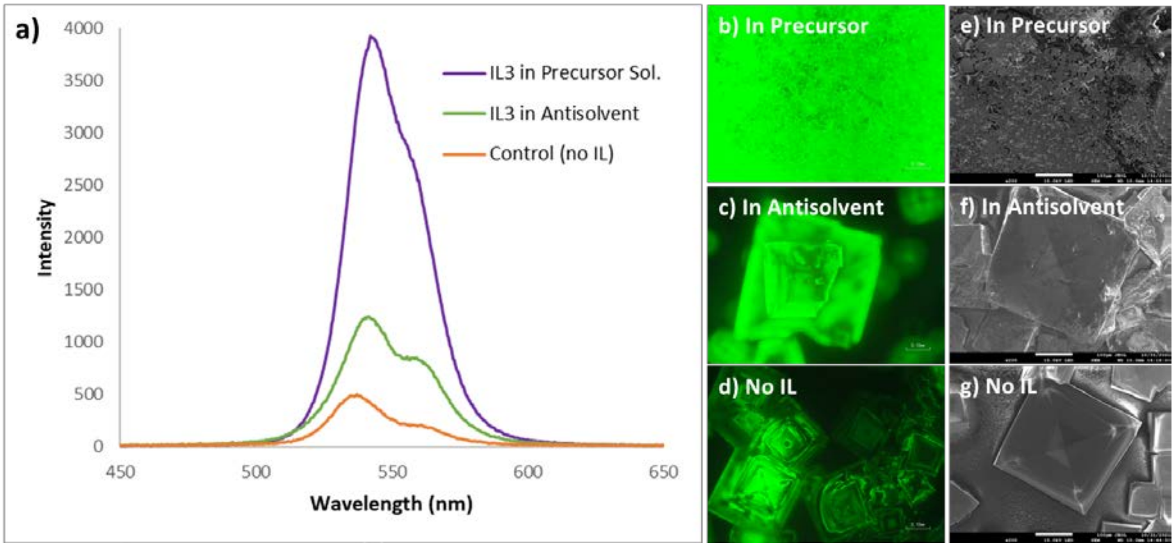

Figure S2: $MAPbBr_3$ prepared via a one-step static deposition method with IL3 added in varying locations: a) PL spectra of all samples; PL imaging of perovskite with IL3 added to (b) precursor solution; (c) antisolvent; (d) non-IL control; (e-g) corresponding SEM images. (scale bar = 100 μm)

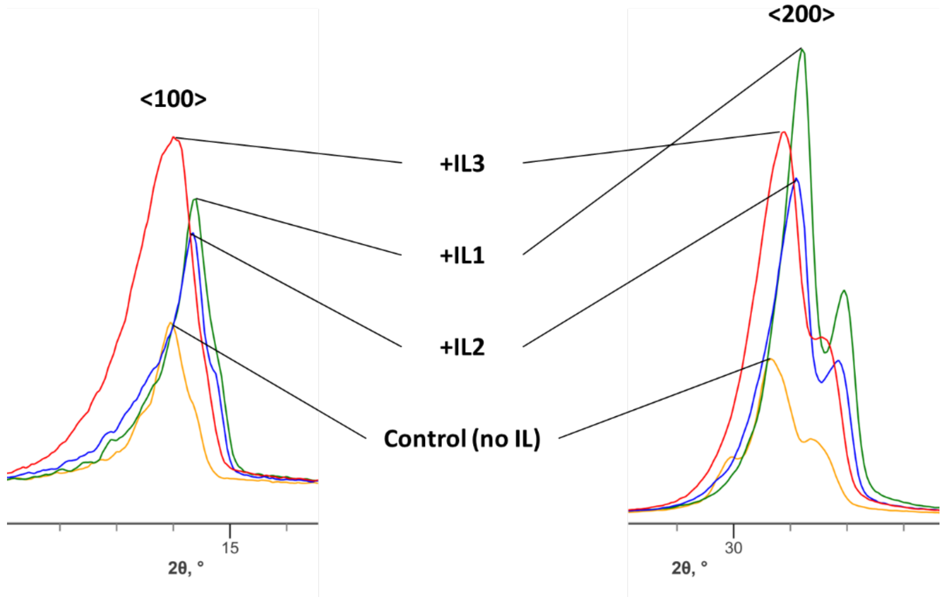

Figure S2: Selected regions of X-ray diffraction patterns of $MAPbBr_3$ perovskite films with IL1 and IL3 used in this study, prepared via a one-step deposition method. Splitting in the <200> peak is a consequence of the Kα1/Kα2 splitting in the Cu x-ray source.

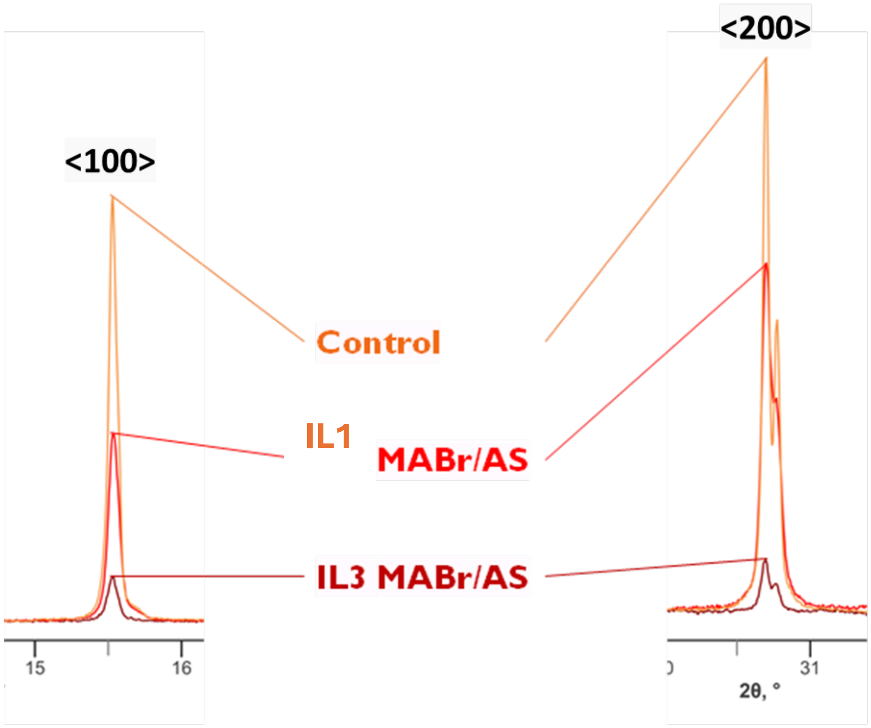

Figure S3: Selected regions of X-ray diffraction patterns of $MAPbBr_3$ perovskite films with IL1 and IL3 used in this study, prepared via a two-step spin coat deposition method. Splitting in the <200> peak is a consequence of the Kα1/Kα2 splitting in the Cu x-ray source.

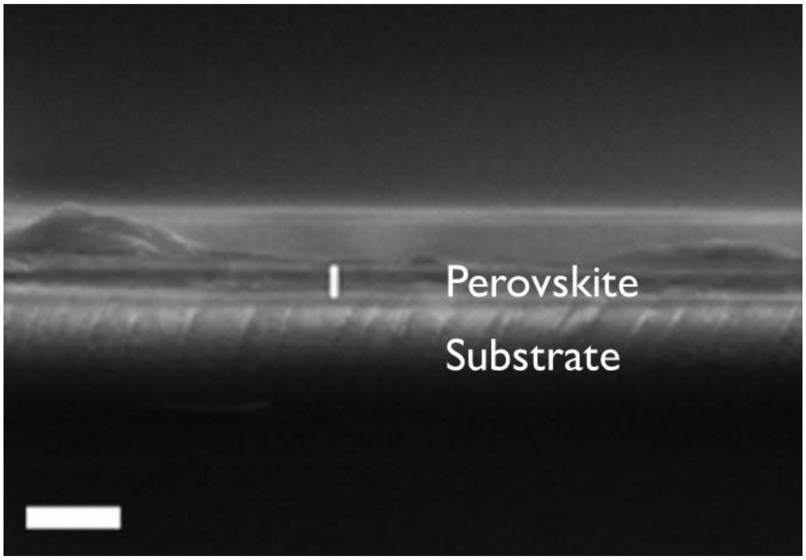

Figure S5: Cross sectional SEM image of $MAPbBr_3$ + IL1 two-step spin coat method. Scale bar is 1 micrometer.

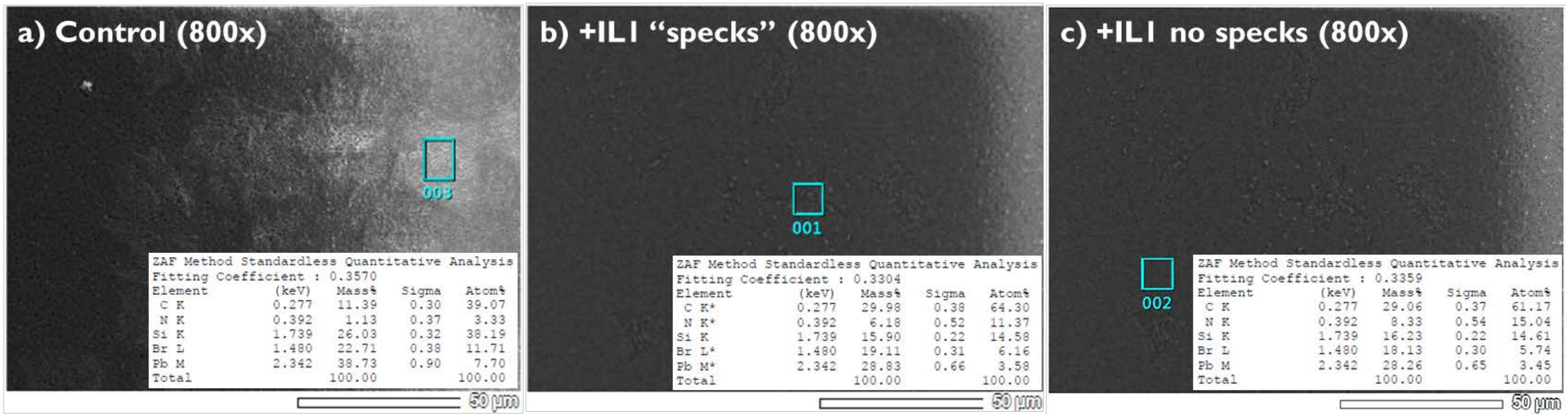

Figure S6: Energy Dispersive X-ray (EDX) spectra of: a) $MAPbBr_3$; b) $MAPbBr_3$+IL1 "specks" region, c) $MAPbBr_3$+IL1 – "no specks" region.

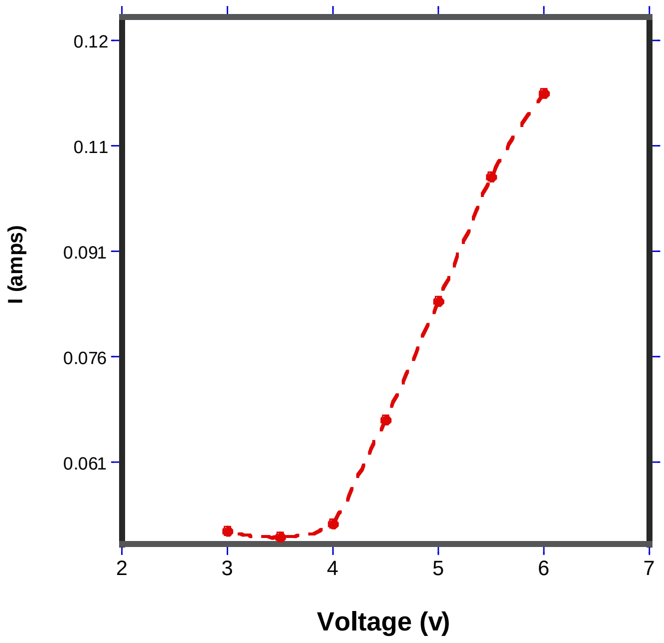

Figure S7: Current-Voltage curve for a typical LED of composition Ag /C_{60} / $MAPbBr_3$ + **IL1** / PEDOT:PSS/FTO.
